# Eclampsia—Purperal Convulsions

**Published:** 1872-10

**Authors:** Alban S. Payne

**Affiliations:** Virginia


### ECLAMPSIA—PURPERAL CONVULSIONS—VARIETY, EPILEPTIC.

BY ALBAN S. PAYNE, M. D., OF VIRGINIA.

Having nothing more interesting on hand at this time, I have concluded to furnish you for publication the following case of "Puerperal Convulsions." Although the case terminated fatally, nevertheless I think it is not devoid of interest to the medical profession:

Mrs. M. L. K——, a very interesting and sensible lady of this neighborhood, who had been married some eighteen months, 42 years of age, '*enciente*' about six months, was suddenly and unexpectedly seized, on the evening of July 28th, with a powerful convulsion. She was taken with a convulsion about six o'clock, P. M. I arrived at 10 o'clock, P. M., same evening, when I received, from her very intelligent husband, the history of her case. Up to the time of her conception she was of ordinary size, with rare mental vigor and judgment. After conception, she had taken on flesh very rapidly, and has become to be quite large and uncommonly fleshy. Her health had been unusually good, occasionally she would complain of sick-headache, but hardly as often as she did before marriage. These periodical attacks of sick-headache were, moreover, hereditary in her family, all her unmarried sisters suffered with occasional attacks of the same.

He had noticed with concern, her rapid increase of flesh, and for a few months back, a certain tumed condition of her face and hands, with œdema of her feet and ankles. He desired and proposed to call in her physician, but his wife said no, I am in better health, have more appetite, feel stronger and have fewer spells of sick-headache than for the last ten years.

The morning before the occurrence of the conversation, she had eaten a small piece of apple-pie, this disagreed with her, causing slight, cholicky pains and diarrhœa, followed by headache, these proved, however, only of a temporary character, and soon passed off, leaving her in usual good health. The morning of the 23d was spent by her sister, who lived near, with her. She attended to her household duties as usual, and appeared cheerful and apparently took much interest in the company of her sister. About six, P. M., some ten minutes after her sister had left for her home, she was seized with a powerful convulsion. I arrived at 10 P. M., in this short space of time, she had had ten convulsions. On my nocturnal journey to her home I had consoled myself with the belief that I was going to an ordinary case of obstetrics; this pleasing delusion was quickly dispelled, for as I passed the thresholed of the front door I caught the glimmer of several agitated countenances as they flitted to and fro, and I heard deep, heavy, stertorous breathing, distinctly, up-stairs. I immediately became calm and collected (although my heart was fairly ready to sink within me), for I had often met this much dreaded and "terrible foe." I hastened up stairs and to her bed-side. She was profoundly comatose, breathing loud, stertorous. Retina insensible to the impression of light, pupils much dilated, face flushed, distorted and of a purplish color, her tongue had been badly bitten during a previous convulsion, and froth and blood commingled together, was issuing from the right side of her mouth. I instinctively felt for my lancet, but upon examination I found her pulse weak, rapid vascillating, oppressed, and she was bathed in a cold, cadaverous, clammy sweat. I made a vaganal examination, and found the paroxysms to be synchronous with the uterine contraction. Os uteri dilated during the contraction to the size of a quarter of a dollar, "the show" was slight, offensive, accompanied by mouches in the loins and abdomen. During this examination there was a large escape of urine. I diagnosed the presentation to be a heel, and at about six months gone. Mrs. K—— might be described as a stout, plethoric woman, of rather coarse muscular fibre, with a short, thick neck, with a preponderance of adiopose tissue. During this vaginal examination, the eleventh convulsion came on, her face became more swollen, more distorted by spasmodic contractions, assumed a darker hue, more of a violet, her eyes are jerked about, the tongue protruded and her under jaw repeatedly closed with force sufficient to seriously wound her tongue were not the handle of a spoon held between her teeth by a faithful attendant. Her mouth is drawn to the right side, and bloody froth is ejected therefrom. The muscles of her body are thrown into violent and irregular action, her limbs are jerked in every direction, and with such power that it is extremely difficult to keep her in bed; her respiration is irregular, and being forced through the foam at the mouth, has a hissing, sibilant sound. Her pulse is very rapid, small, almost imperceptable at the wrists. The body, during the paroxysm, participates in the violet color of the face. After a period varying from ten to thirty minutes, the convulsion gradually subsides, when she either passes into a quiet state of total insensibility with sibilant or stertorous breathing, or with a restless throwing about of the body and extremeties. The interval is about thirty minutes, and is preceded by uterine contraction, with slight dilitations of os uteri. Every circumstance seemed to teach me that her last final hour was near at hand, and the slight remaining chance, was to rid the uterus of its distasteful load as soon as practicable. I would bleed freely, but it is impracticable, in her present condition. I therefore, abandon all idea of venesection for the time, and prescribe ext. coly. comp., grs. x, sol. mur. hydrag, grs. x, pulv. ipecac, grs. ss., stimulating salt enema, emp. cantharides to nape of neck and mustard plasters to inside of thighs, and epigastrium, and hastily dispatched a messenger for Drs. Stribling and Marshall, sending also for a bottle of chloroform and a vial of croton tiglium. One o'clock, P. M., Dr. Marshall has arrived. No action from the medicine. Convulsions continue every thirty minutes, muscular convulsions still powerful. R—Purgative to be repeated, stimulating enema every two hours; lin. of oil of turpentine and chloroform rubbed from ligamentun nuchæ to os coxygis. We would gladly bleed, but cannot do so. During the interval we placed her twice fully under the influence of chloroform; from this there is no improvement in either the force or frequency of the convulsions. Four o'clock, P. M., no change in her condition. R.—Ice to her head with croton tiglium, gtts. jii.

July 24th, 7 o'clock, A. M., not much change. Convulsions less frequent. Every two hours, a shade lighter. No action from bowels or from enema. Suppression of urine (this I regret, as I intended to procure some to test). Bowels began to rumble, as if medicine would act. No improvement in the appearance or sensibility of the patient. Eight o'clock, A. M., pulse is rising, no other noticeable improvement. Nine o'clock, A. M., Dr. Stribling has joined us, patient in "*statu quo.*" Will bleed as soon as possible, and then resort to artificial dilitation of os uteri. At 10:30, she was freely and skillfully bled by Dr. Stribling, and at 11, o'clock, A. M., we commenced with our artificial dilitation of the os, each of us taking it by turns, and relieving each other as our hands gave out. At 2 o'clock, P. M., Dr. Stribling, after the most persistant effort, so far overcame the refractory uterus as to introduce his whole hand into the uterine cavity, grasped the heel and quickly and skillfully delivered the patient of a still-born child at about six months in uteri. The placenta also had to be delivered by the hand. At 2 o'clock, P. M., our patient looks better, seems to be more comfortable. Some very slight evidence of returning sensibility, frowns if you pinch her, has been seen to brush a fly away that had lit upon her face. Medicine has acted copiously. No spasm since 7 o'clock, A. M. Takes her medicine better. Four o'clock, P. M., medicine has acted again, discharges look dark and bilious. Suppression of urine still continues. A distressing cough has set in. She continued about in this condition until 7 o'clock, P. M., when the medicine again acted copiously, this time rather watery in its character; cough more harrassing. R.—Sul. morphine, grs. ¼. Having given the morphine, and having all gone through great fatigue, we all lay down in the parlor. At 10 o'clock, P. M., I am aroused by a little noise up-stairs. I hastily go up to see from whence it proceeds. Morphine has had no effect; the cough truly distressing, and I find engrafted upon the loud, stertorous breathing, that peculiar but never to be forgotten rattle (the death-rattle). I knew she must die, and thus

> "The young, the beautiful the true,
> In a few short hours would be
> A thing o'er which the raven flaps her funeral wing."

From this hour, evincing great tenacity of life, she gradually sank, and at 4 o'clock, P. M., July 25th, she had ceased to breathe.

Various and very obscure have been the explanations of the causes of puerperal convulsions. Dr. Locock thus enumerates them: "The immediate causes of puerperal convulsions are often very obscure. They appear sometimes to depend upon a loaded state of the brain; at other times the brain appears to be influenced by distant irritation, either in the uterus or digestive organs; and again, in some cases, puerperal convulsions are induced, apparently, by a peculiar irritability of the nervous system. It has been remarked that there has been a greater disposition to puerperal convulsions in those patients who have been, in early life, subject to convulsive attacks, partially of an epileptic character; and also, in those who have suffered similarly in former labors, and have omitted those measures usually employed as precautions. That the uterine organs are in some way partially implicated, is evident from the convulsions being of a character which may be said to be peculiar to the state of either pregnancy or parturition. The immediate attack may be brought on by a loaded or disordered stomach, or by food however small in quantity, of an indigestible kind. Some substances. (shell-fish, for instance), have been found very frequently to induce convulsions in the puerperal state, when at other times they may be taken by the same individual with perfect impunity. A sudden fright, afflicting intelligence, or any unexpected or depressing mental emotion, may excite the paroxysm; hence, it has been long remarked, that unmarried women are more particularly likely to be sufferers from convulsions, from the shame and distress under which their children are usually born. The violent straining caused by labor pains, from disturbance of the frame by the earlier uterine contractions, causing a temporary rush of blood to the head, will sometimes bring on convulsions. The application of Dr. Marshall Hall's theory, however, by Drs. Thompson, Murphy, and Tyler Smith, has thrown much light upon the matter. The former gentleman insists that no injury to the cerebrum or cerebellum can cause convulsions so long as the true spinal system is not involved, in which Dr. T. Smith agrees with him. He then states that the proximate cause of puerperal convulsions consists in a morbid irritation of the true spinal system, more especially of the medulla oblongata propagated to it from mucous surfaces, through the incident nerves of the excito-motor system. Dr. Murphy enumerates, among the proximate causes, morbid irritation of the uterus from hyperæmia or anæmia and morbid irritation of other organs, and regards the whole as a beautiful illustration of the reflex nervous function; the puerperal nerves that supply the affected organ rapidly communicating their irritation to the spinal system, which, as an excito-motor centre, radiates the irritation over the whole of the voluntary muscles and the muscles of respiration. Even the involuntary muscles, as the heart and uterus, do not escape. Dr. Tyler Smith, in his admirable work, has entered into a most elaborate investigation of the causes of convulsions; after which he observes: In conclusion, to give a summary of the whole subject, the true puerperal convulsion can only occur when the central organ of the system, the spinal marrow, has been acted on by an excited condition of an important class of its incident nerves, namely, those passing from the uterine organs to the spinal centre, such excitement depending on pregnancv, labor or the puerperal state. While the spinal marrow remains under the influence of either of these stimuli, convulsions may occur from two series of the causes: those acting primarily in the spinal marrow, or centric causes; and secondly, those affecting the extremeties of its incident nerves; causes of eccentric or peripeheral origin:

I. Causes acting immediately on the central organ. 1. Pressure exerted on the medulla oblongata by congestion, coagula, nervous effusion within the cranium. 2. Loss of blood. 3. Morbid elements in the blood. 4. The influence of emotion.

II. Causes acting on the extremeties of excitor nerves: 1 Irritation of the incident spinal nerves of the uterus and uterine passages. 2. Irritation of excitor nerves within the cranium. 3. Irritation of the incident spinal nerve of the rectum. 4. Irritation of ovarian nerves. 5. Irritation of the gastric and intestinal branches of the pneumogastric nerve. 6. Irritation of the incident spinal nerves of the bladder. 7. As probable causes may be enumerated: irritation of the cutaneous nerves of the mammæ and of the hepatic and renal branches of the pneumogastric. Though the subject admits of this division, several causes may act together and centric and eccentric may be in operation at the same time. I have made no attempt at a division into predisposing and exciting, proximate and remote causes, as other authors have usually done, because it is evident that a cause which, in one case is the exciting or proximate, may in another be the predisposing or remote cause. Subsequently, Dr. Tyler Smith endeavored to explain the operation of the causes and to trace the gradual progress from the slight commencement up to the completion of the convulsive paroxysms; but the investigation, though able and full of interest, is too long for quotation. Among the most exciting causes are usually enumerated: intemperance in eating and drinking; mental emotions; fright, as in the case related by Denman, of a lady going on a party of pleasure, and whose carriage broke down; she was near the time of her lying in, and was very much frightened, though she received no injury; labor came on, preceded by convulsions, and she died undelivered.

Mr. Robbs has related a case in which the convulsions seem to have been caused by the irritation of worms; at least they ceased on the expulsion of two large lumbrici. Atmospheric influence, according to M. Duges, appears to have some peculiar effect in producing the disease, so that it assumes the character of an epidemic. Dr. Ramsbotham's observation confirms the same.

Albuminuria cannot be overlooked as an exciting cause. Hamilton and Demauet first stated that puerperal convulsions were liable to be preceded by anasarca, and their observations were confirmed by the highest authority. Dr. Simpson and Dr. Lever were the first to connect this dropsy with that condition of the kidney which gives rise to albumen, and since their time, the researches of Colier and Bouchut, Roger, Depaul, Cazeaux, etc., have confirmed and extended their observations. Dr. Cormick has published an excellent paper on the connection between renal congestion and puerperal convulsions. He considers that in many cases the latter are the toxicological results of non-elimination of the excretions of the blood, and that in the greater majority of cases their non-elimination depends upon renal congestion, caused by the pressure of the gravid uterus. My experience teaches me to incline strongly towards the opinion so concisely stated by Dr. Cormick. Pressure of the gravid uterus producing congestion of the kidney, its secretion becomes morbid, thus non-eliminated from, but rather conveyed into, the system, becomes a blood poison, and "puerperal convulsions," rather of the variety hysterico epileptic tetanic or apoplectic is the result. No one, I presume, will deny the dire influence of predisposing, as well as exciting causes in the production of eclampsia, and most other diseases. I think, therefore, when a young female, pregnant with her first child, is seen to grow robust rapidly, with tumid hands and face, and with small lower extremeties, it is sound practice to take blood from her arm, give some diuretic medicine and clear out the alimentary canal by a brisk bilious purge. I am satisfied, in my own mind, had this been done in Mrs. R——'s case, on the morning of the 23d, the convulsions would not have supervened. I cannot close this paper without tendering to Drs. Robert M. Stribling and Jacquelin A. Marshall my warmest thanks for their very valuable and timely aid. I found in them all I could ask, kind, attentive, intelligent, and fully up with their profession.

